# Clinical and molecular report of novel *GALC* mutations in Moroccan patient with Krabbe disease: case report

**DOI:** 10.1186/s12887-015-0490-9

**Published:** 2015-11-13

**Authors:** M. Zerkaoui, I. Ratbi, B. Castellotti, C. Gellera, J. Lyahyai, Y. Kriouile, A. Sefiani

**Affiliations:** Centre de Génomique Humaine, Faculté de Médecine et de Pharmacie, Université Mohammed V, Rabat, Morocco; Département de Génétique Médical, Institut National d’Hygiène, Rabat, Morocco; Unità di Genetica delle Malattie Neurodegenerative e Metaboliche, Dipartimento di Diagnostica e Tecnologia Applicata, Fondazione IRCCS –Istituto Neurologico Carlo Besta, Milan, Italy; Unité de Neurologie Pédiatrique de Maladies Métaboliques, Service de Pédiatrie IIA, Hopital d’Enfant, Faculté de Médecine et de Pharmacie, Université Mohammed V, Rabat, Morocco

**Keywords:** Krabbe disease, *GALC* gene, Galactocerebrosidase, Globoid cell leucodystrophy

## Abstract

**Background:**

Krabbe disease (KD) or globoid cell leukodystrophy is an autosomal recessive lysosomal disorder, which affects metabolic and neurologic systems. This pathology has different forms. Infantile onset is about 85 % to 90 % of individuals with Krabbe disease. Disorder’s onset is characterized, in early childhood, by hyperirritability, psychomotor deterioration associated to episodes of fever. To date, all reported cases have been attributed to mutations in galactosylceramidase gene (*GALC* gene) that encodes an enzyme which degrades galactosyl-sphingolipids (galactosylceramide, psychosine), essential in myelin production. A child compounded with two new mutations in the *GALC* gene was detected.

**Case presentation:**

An eleven month old male child of Moroccan origin presented to our genetic consultation with severe symptoms that included hypotonia, fever, vision loss and feeding difficulties. He was suffering from the 4th month of life. Krabbe disease was suspected. Galactocerebrosidase deficiency was confirmed by biochemical analysis. DNA sequencing revealed a novel heterozygous compound mutation in *GALC* gene. The child was compounded with two mutations c.860G > A; p.Cys287Tyr and c.1622G > A; p.Trp541*.

**Conclusion:**

These new mutations could affect GALC structure and therefore its function. The identification of these mutations and their associated phenotypes are important to predict the prognosis and to confer to families an adequate genetic counseling.

## Background

Krabbe disease (KD) (also known as globoid cell leucodystrophy GCL OMIM #245200) is a rare inherited metabolic and neurodegenerative disease, which is pathologically not completely elucidated. This autosomal recessive lysosomal disorder affects the white matter of the central and peripheral nervous systems. This is the result of deficiency of the lysosomal enzyme beta galactocerebrosidase (galactosylceramidase, GALC) or, in very few cases, it is due to lack of activin saposin A (sphingolipid activator protein) [1–3]. The deficiency of GALC impairs the degradation of a major myelin lipid, galactocerebroside and that of a parent cytotoxic compound, galactosylsphingosine also called psychosine [4]. The excess of galactosylceramide elicits the formation of multinucleated macrophages, the globoid cells. Progressive accumulation of psychosine can explain the prominent death of oligodendrocytes and myelination arrest, and contributes to progressive demyelination [5].

**Fig. 1:**
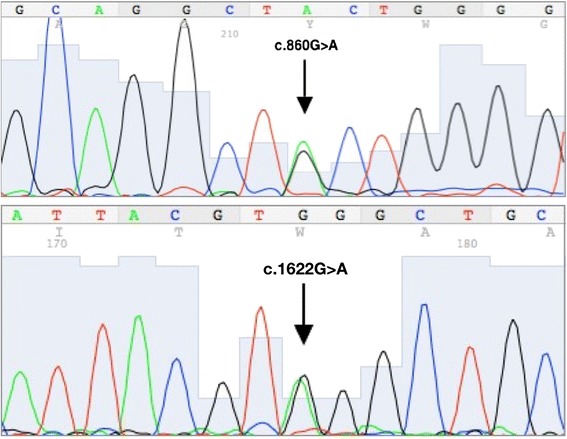
DNA sequencing of the patient showed two novel *GALC* gene mutations, c.860G>A inherited from his mother and c.1622G>A inherited from his fatherᅟ

In 1916, Danish Neurologist Knud Krabbe described for the first time a globoid cell leucodystrophy in three families [6]. The prevalence of the disease is today evaluated at approximatively 1/100000 births with wide variation between countries.

Krabbe disease forms vary in age of onset and clinical course. The typical infantile form is a severe, rapidly progressive and demyelinating disease. First symptoms appear before the age of 6 months including hyperirritability, stiffness, hyperactive reflexes and episodes of elevated temperature followed by psychomotor deterioration, seizures, spasticity, and vision loss. However, the clinical outcome is most variable in late onset (i.e. juvenile and adult forms of globoid cell leucodystrophy). As a matter of fact, muscle weakness, vision loss and arrest of intellectual development are consistently observed [2, 7].

All forms are caused by mutations in the *GALC* gene. This gene, located on chromosome 14q31, encodes for the beta galactocerebrosidase protein, which has a hydrolase function which is critical for glycosphingolipid catabolism. More than 130 mutations have been cataloged in Human Gene Mutation Database (HGMD) so far, at least 128 of which being reported as the cause of KD.

According to our extensive literature review, and to the best of our knowledge, this is the first case of KD to be reported in Morocco. We describe therefore the first identification of *GALC* mutation in a Moroccan family.

## Case presentation

An eleven month old male child, from northern Morocco, born to unrelated parents was referred to our medical genetics consultation with low level of galactocerebrosidase; molecular analysis was necessary to confirm the diagnosis of Krabbe disease.

The pregnancy had been medically followed, and no complications were reported. Weight at birth was 3300 g; the mother presenting no history of drug ingestion nor phytotherapy. There was no family history of congenital anomalies. The infant was the unique to a 24 years old mother and 41 years old father.

On evaluation, both of parents were found normal. From birth to 4 month, the child had a normal development and normal feeding. From then, his development plateaued and rapidly regressed. Simultaneously, the child presented episodes of fever and pulmonary infection. The parents observed that the infant was very irritable. Medical consultation revealed a generalized hypertonia, increased deep tendon reflexes and arching. Gradually, the patient gradually lost mobility of his members. Since then, he presents a generalized hypotonia. Hearing appeared normal, and vision was lost. This symptomatology prompted the pediatric neurologist to require galactocerebrosidase level. The result revealed a subnormal level 0,3 nmol/mg protein per h, with normal values being > 0.7 nmol/mg protein per h. These findings suggested a molecular analysis of *GALC* gene. Genomic DNA for the 3 family members was extracted from peripheral blood leukocytes with a Qiagen kit according to the manufacturer’s instructions (QIAGEN, Germany). The quality and quantity of the DNA were checked by A260/A280 using a Nanodrop spectrophotometer (Nanodrop Technologies).

The clinical data collection and genomic analysis was approved by the institutional ethics committee, and the two parents provided their written informed consent.

The general condition of our patient worsened increasingly. A gastrostomy tube was placed due to feeding problems. The child became progressively unable to manage his oral secretions and succumbed to aspiration pneumonia at 14 months of age.

## Discussion

The major form observed in Krabbe Disease is the infantile form (85–90 %), characterized by rapidly progressive neurologic deterioration and death before the age of two [8]. Children with such form appear to be healthy for the first few months of life but show extreme irritability, hypersensitivity to the external environment, stiffness of the limbs, and episodic fever with no apparent reasons. The psychomotor regression (hypertonicity, extended and crossed legs, flexed arms and backward head) progresses to a decerebrate posture with no voluntary movement [2]. The onset of symptoms and clinical course can differ even among siblings. The late infantile form (onset from 7 months to 12 months) and the juvenile form (onset from 1 to 10 years age) are characterized by spastic tetraparesis, cerebellar ataxia, optic atrophy, mental retardation and cognitive decline. Onset may also occur after 20 years in the adult form. Some individuals may remain stable for long periods, while others show a continuous decline in vegetative state and die. Especially in the adult phenotype, no clear genotype-phenotype relationship is demonstrated [2, 5].

Galactocerebrosidase (GALC) enzyme activity deficiency is observed in almost all cases of Krabbe disease [9]. In the literature, no consistent correlation has been observed between residual GALC enzyme activity measured in leukocytes or cultured skin fibroblasts, and age of onset or disease course [8, 10].

Different kinds of mutations have been described such as missense/nonsense, small or gross deletion, splicing, insertions and duplications [2]. Many different mutations affecting each of the 17 exons have been reported; the majority of which are missense mutations leading to the infant form of Krabbe disease [8]. A 30-kb deletion, accounts for approximately 35 % of mutant alleles in individuals of Europe and the USA [11, 12]. This large deletion appears in the homozygous state or in the compound heterozygous state along with another mutation known to cause infantile Krabbe disease.

In our clinical case, The onset of the disease was early in the 4th months of life. The disease’s evolution has been so fast that when we examined the patient on the 5th month, he was entirely hypotonic and already experienced feeding difficulty. Proband’s GALC enzyme activity was slightly decreased. This evolution is compatible with infantile form, which motivated us to seek the deletion of 30Kb by real time PCR. Once we observed that there was no such deletion, we opted for the complete sequencing of the gene. This method revealed that our patient was heterozygous compound for the mutations c.860G > A (p.Cys287Tyr) and c.1622G > A (p.Trp541*) (Fig.1).

The first mutation is a single nucleotide substitution (c.860G > A) in exon 8 of the *GALC* gene. The result showed a substitution of cysteine at position 287 to tyrosine (p.Cys287Tyr). The cysteine in that position is maintained in the 7 species reported in Multiz Alignment and Conservation of the UCSC Genome Browser. PolyPhen and SIFT predicted that this substitution was respectively “probably damaging” (with score of 0.999) and “deleterious” (with score of 0.01). This mutation was found in the proband’s mother for which she was heterozygous.

The second novel mutation c.1622G > A (p.Trp541*) found in our patient is also a single nucleotide substitution in exon 14 which causes a premature termination of the GALC protein. The child inherited this mutation from his father who was heterozygous for the mutation c.1622G > A.

It has been noted that usually *GALC* mutations in the infantile form occurred in the central domain, while in the adult form the N- or C-terminus is mutated [2, 13, 14].

More so, some mutations clearly result in the infantile type if found in homozygous state or in compound heterozygous state with another severe mutation, though it is difficult to predict the phenotype of novel mutations or mutations found in apparent heterozygous state.

Some common polymorphisms influence enzyme activity and may be responsible for a pseudodeficiency state, particularly when in compound heterozygosity with disease-causing allele [12].

A high frequency of polymorphic changes on apparent disease-causing alleles complicates the interpretation of the effects of mutations.

Although our patient was a compound heterozygote for the disease, he presented a slightly decreased level of galactocerebrosidase.

Molecular analysis of the proband has allowed for an appropriate genetic counseling for the family.

## Conclusion

We presented in this paper the first identification of a *GALC* mutation in a Moroccan family. It is a novel double heterozygous compound mutation in a Moroccan child, who presented infantile KD form with decreased GALC enzyme activity. These mutations may help to enrich the *GALC* pathogenic mutation database and increase public awareness of Krabbe disease in Morocco.

## Consent

Written informed consent was obtained from the parents of the patient for publication of this case report and for any accompanying images. A copy of the written consent is available for review by the editor of this journal.

## Abbreviations

KD: Krabbe disease
GALC: GAlactocerebrosidase
PCR: Polymerase chain reaction
